# What are the effects of psychological stress and physical work on blood lipid profiles?

**DOI:** 10.1097/MD.0000000000006816

**Published:** 2017-05-05

**Authors:** Seyedeh Negar Assadi

**Affiliations:** Department of Occupational Health Engineering, School of Health, Social Determinants of Health Research Center, Mashhad University of Medical Sciences, Mashhad, Iran.

**Keywords:** lipid disorder, physical activity, stress, work

## Abstract

Blood lipids disorders are prevalent in the world. Some of their risk factors are modifiable such as mental and physical stress which existed in some places such as work environment.

Objective of this study was to determine the effects of psychological and physical stress on the lipid profiles. It was a historical cohort study. The people who were employed as general worker were participated. The study was conducted with flexible interview for getting history, lipid profile examination, and a checklist including occupational and nonoccupational risk factors and using the health issues. According to the type of stress exposures, the study population was divided into 5 groups. Groups were followed for lipid profiles. These groups were exposed to psychological stress, physical stress or both of them; mild psychological stress (group 1), mild physical work without psychological stress (group 2), mild psychological stress and mild physical work (group 3), moderate physical work without psychological stress (group 4), and heavy physical work without psychological stress (group 5). Data were analyzed with SPSS 16. ANOVA, *χ*^2^, and exact test were calculated with considering *P* < .05 as significant level. Relative risks were calculated with confidence interval 95%. The means of lipid profiles were in normal ranges. The relative risks for triglycerides more than 200 mg/dL was 1.57 (1.02–2.42) and low density lipoprotein (LDL) more than 130 mg/dL was 14.54 (3.54–59.65) in group 1. The relative risks for high density lipoprotein (HDL) less than 45 mg/dL was 14.61 (8.31–25.68) in group 1 and 16.00 (8.30–30.83) in group 3. After multinomial logistic regression they had significant differences. Psychological stress was a risk factor for lipid disorders, and suitable physical activity was protective in this situation.

## 1 Introduction

Lipid disorders are prevalent in the world.^[1]^ Some of their risk factors are modifiable such as mental and physical stresses in some situations like workplaces.

The main etiology of lipid disorders is genetic factor and family history that is not changeable. In recent decade researchers have worked on risk factors for lipid disorders.^[1]^ Hypertriglyceridemia, hypercholesterolemia, and related lipid disorders are very common, their prevalence are between 20% and 50% in different populations.^[1]^ There are a few studies that showed the role of environmental risk factors for dyslipidemia besides nutritional conditions.^[1,2]^

Psychological stress had effects on human body especially on some specific organs and parameters and physiological parameters too, lipid profile was one of them. Physical stresses caused by physical works could be affected lipid profiles too. Night shift work could be a risk factor for hyperlipidemia but the background of well-being is important in this situation.^[2,3]^

Researchers reported lipid disorders; related to job stress in professional drivers. Their study showed the effects of stress on triglycerides, low density lipoprotein (LDL), and high density lipoprotein (HDL).^[4]^ Scientists showed the relationship between job stress and dyslipidemia including total cholesterol and LDL and decreased HDL.^[5]^

Researchers studied the association between the occupational stress and hypertension, type 2 diabetes mellitus, lipid disorders.^[6]^

Other researcher showed the cardiovascular disease and its risk factors in law enforcement personnel.^[7]^ Another study demonstrated the association between job stress and combined dyslipidemia among workers.^[8]^ There are also some studies about the dyslipidemias in female law enforcement officers and railway workers, and male aircrew personnel.^[9–11]^

Some studies showed lipid disorders in people with jobs that had psychological stress.^[12–14]^

Researchers demonstrated the effectiveness of wellbeing, preventive methods, and treatment on lipid disorders.^[15–17]^ Night shift work was reported as a risk factor for cardiovascular disorders in different jobs,^[18–20]^ which are common in the society.^[21,22]^ Chemicals such as carbon disulfide was also introduced as a cardiovascular risk factor.^[23,24]^ All together some studies have showed the effects of work stress on health and wellbeing.^[25–28]^

Objective of this study was to determine the effects of psychological and physical stress on lipid profiles.

## 2 Materials and methods

Study design and target population; it was a historical cohort study, which was performed on people who were employed as general workers during 2005 to 2016. The main aim was to compare the effects of psychological and physical stress on participants’ lipid profiles. Data were collected with flexible interview, physical examination, and a checklist including history, measurement of lipid profile and risk factors and using the data from health issues. According to type of exposures the study population was divided into 5 groups. Groups were followed for lipid profiles. These industries had not another risk factor for lipid profile changes. They were not used carbon disulfide, they had low to moderate fat in nutrition. All of them had shift work.

Simple random sampling method was used with α = 0.05, power = 90, P1 = 25%, and P2 = 50%, the calculated study population was 1000 for each group (5 groups), and 5000 in total.

The inclusion criteria were people who worked in general working with at least 5 years work experience in the same work. The exclusion criteria were having the hyperlipidemia and related diseases before beginning this job and having the positive family history in lipid profile disorders and anxiety disorders.

The validity and reliability of checklist were checked with specialists’ opinions and also with performing a pilot study with correlation coefficient 90%. The participants were interviewed by author using a checklist. The results of blood examinations in periodic examination were taken and body mass index (BMI) was calculated. The level of cholesterol in total and ingredients (LDL and HDL) and triglyceride were important for researcher. These values were high risk; BMI was equal and more than 30 kg/m^2^, triglycerides was equal and more than 200 mg/dL, total cholesterol was equal and more than 200 mg/dL, LDL was equal and more than 130 mg/dL, and HDL was equal and more than 45 mg/dL.

### 2.1 Exposure assessment

Two types of physical and psychological stress were assessed in this study and 5 groups with different exposures were evaluated: mild psychological stress (group 1), mild physical work without psychological stress (group 2), mild psychological stress and mild physical work (group 3), moderate physical work without psychological stress (group 4), and heavy physical work without psychological stress (group 5).

Group 1: workers with more than 1% to 25% of total grade in work environmental scale and modified standard stress scale. Group 2: workers with mild physical work without psychological stress. Group 3: workers with more than 1% to 25% of total grade in work environmental scale and modified standard stress scale and mild physical work. Group 4: workers moderate physical work without psychological stress. Group 5: workers with heavy physical work without psychological stress.

Job stress was assessed with work environmental scale and modified standard stress scale; there were 10 items with 0 to 10 grades. Items were in organizational (change, coworkers, supervisor relationships), career development (achievement, improvement), role (ambiguity, conflict), task (under or over load), and environmental fields. Stress were recognized with more than 1% to 25% of total grades as mild level, and the severity of physical work was assessed with standards aerobic tests (McArdle step test) and calculated metabolic equivalent tasks or metabolic equivalent of tasks (METs) with according to VO_2_ max (mL/kg/m) at the preplacement of participants; preplacement examinations results were used for physical stress determination. METs less than or equal to 3 indicates mild activity, between 3 and 6 shows moderate activity and more than 6 declares a heavy work. Other work exposures were kept in the standard levels.

The researcher determined the stress level according to work environmental scale and modified standard stress scale. By using of blood examinations were done in periodic examinations the relation between the job risks and lipid profiles were showed.

For statistical analysis, data were analyzed with SPSS 16. *χ*^2^, exact test, ANOVA, and regression were used to compare qualitative and quantitative variables, *P*-value less than .05 was considered for significant levels and relative risks were calculated with confidence interval 95%.

### 2.2 Ethical consideration

This study, involving human participants, was done in accordance with the ethical standards and with the 1964 Helsinki declaration and comparable ethical standards and was implemented by getting consent that was obtained from all the participants. The researcher used from preplacement for physical tests and periodic examinations for lipid profiles in the industries.

## 3 Results

The study participants were divided into 5 groups based on exposure to physical or psychological stresses. The age, work duration, total cholesterol, and HDL showed significant differences between study groups (*P* < .05). They were male workers and had not smoking. They had rotating shift work and low to moderate fat food.

Participants in group 4 (moderate physical work) had the highest age, work duration and BMI. Triglycerides, LDL were the most in group 5 (heavy physical work) and HDL was the least in this group too. Total cholesterol had the highest level in group 3 (mild psychological stress and mild physical work). The results of blood test are demonstrated in Table 1 (*P* < .05).

**Table 1:**
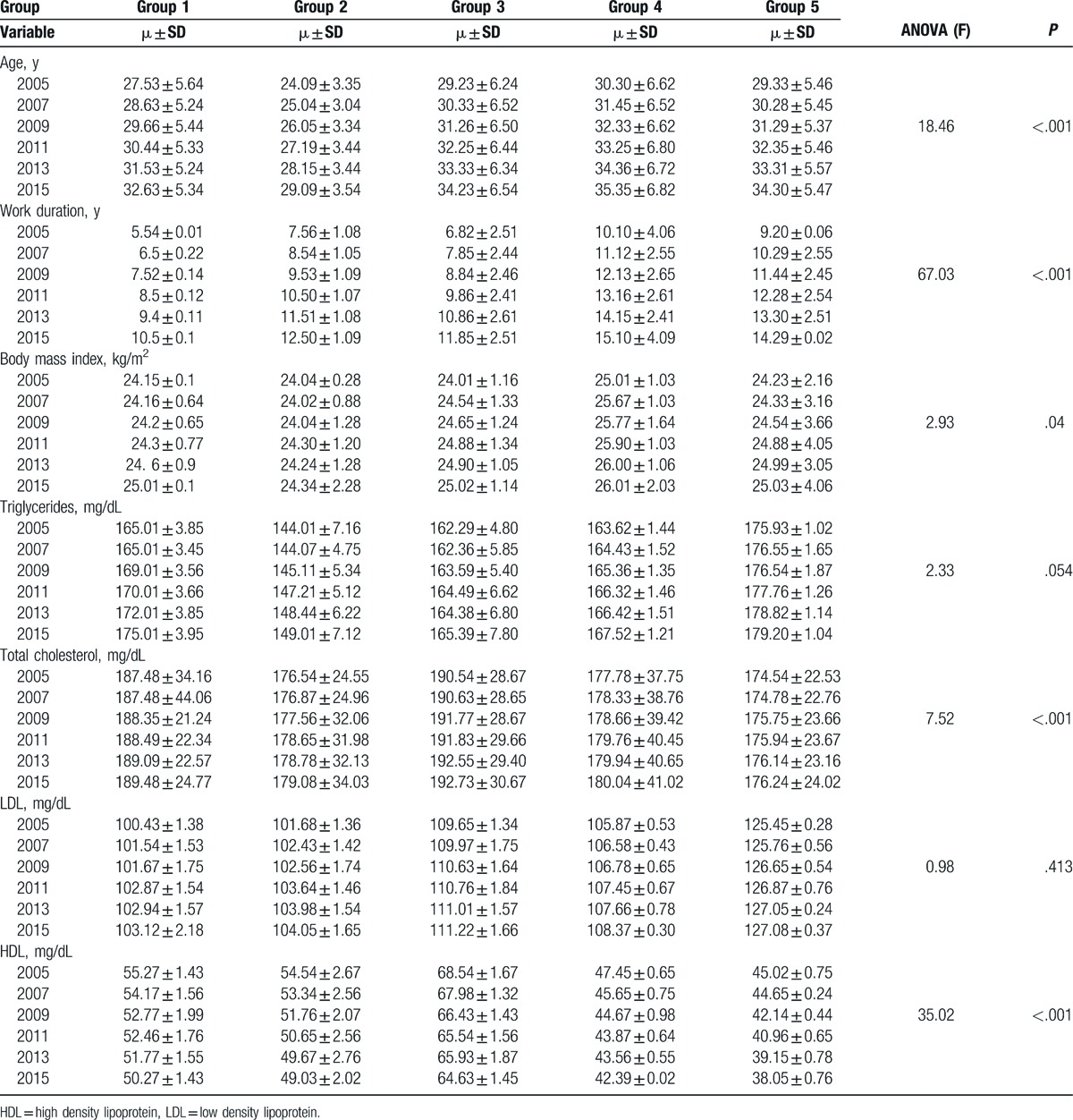
Means of risk factors amounts and comparison between 5 groups (*P* < .05).

The highest number of persons with BMI more than 30 and total cholesterol more than 200 was in group 4. The highest number of workers with triglycerides more than 200 and HDL less than 45 was found in group 5. The most number of participants with LDL more than 130 were in groups 5. These items are demonstrated in Table 2 (*P* < .05).

**Table 2:**
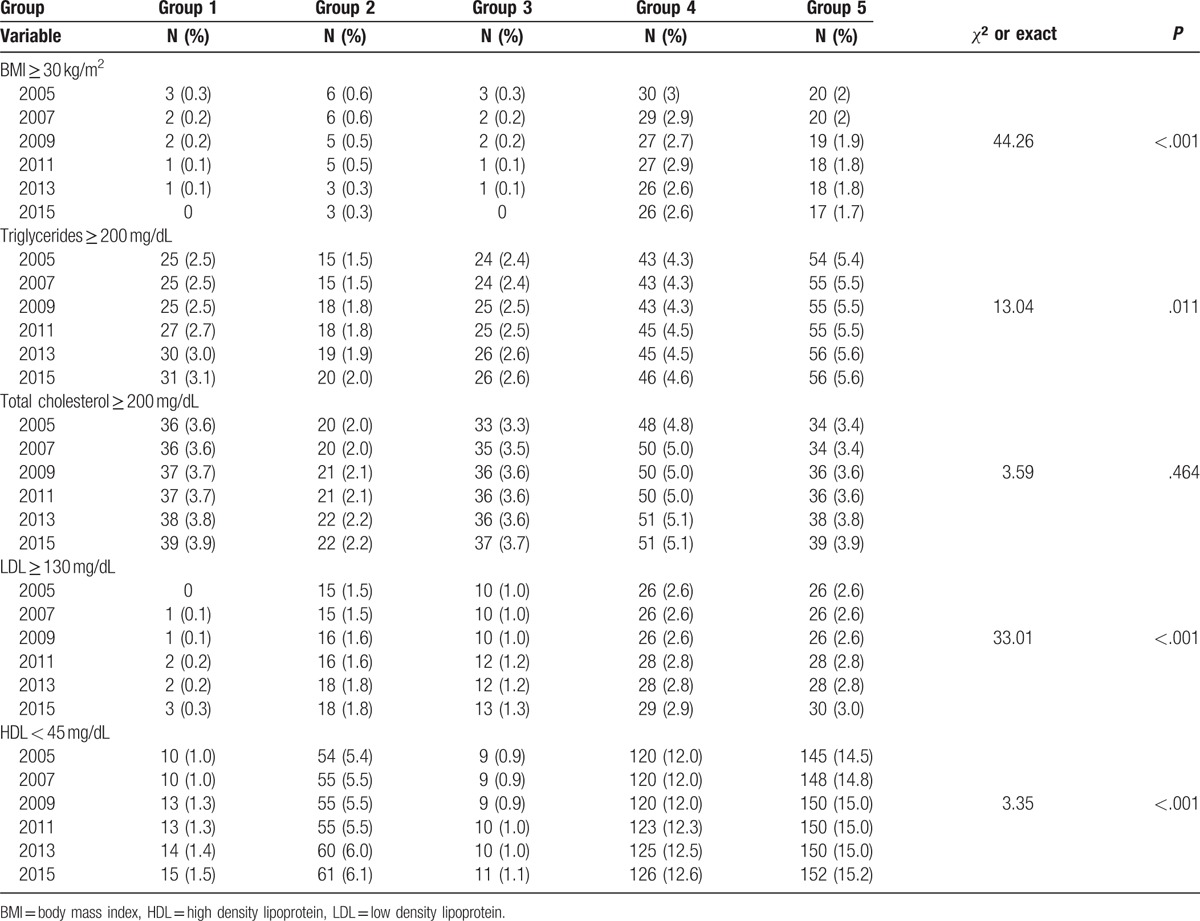
Frequencies of risk factors and comparison between 5 groups (*P* < .05).

After deleting the effect of BMI and age with regression, the relative risks for triglycerides more than 200 mg/dL was 1.57 (1.02–2.42) and for LDL more than 130 mg/dL was 14.54 (3.54–59.65) in group 1 (mild psychological stress). The relative risks for HDL less than 45 mg/dL were 14.61 (8.31–25.68) in group 1 and 16.00 (8.30–30.83) in group 3. In groups 2 and 4 the relative risks of LDL more than130 mg/dL and HDL less than 45 mg/dL were below one. The relative risks in group 5 was below one; 0.62 (0.387–1.00), 0.150 (0.104–0.215). Table 3 shows the relative risks in different groups.

**Table 3:**
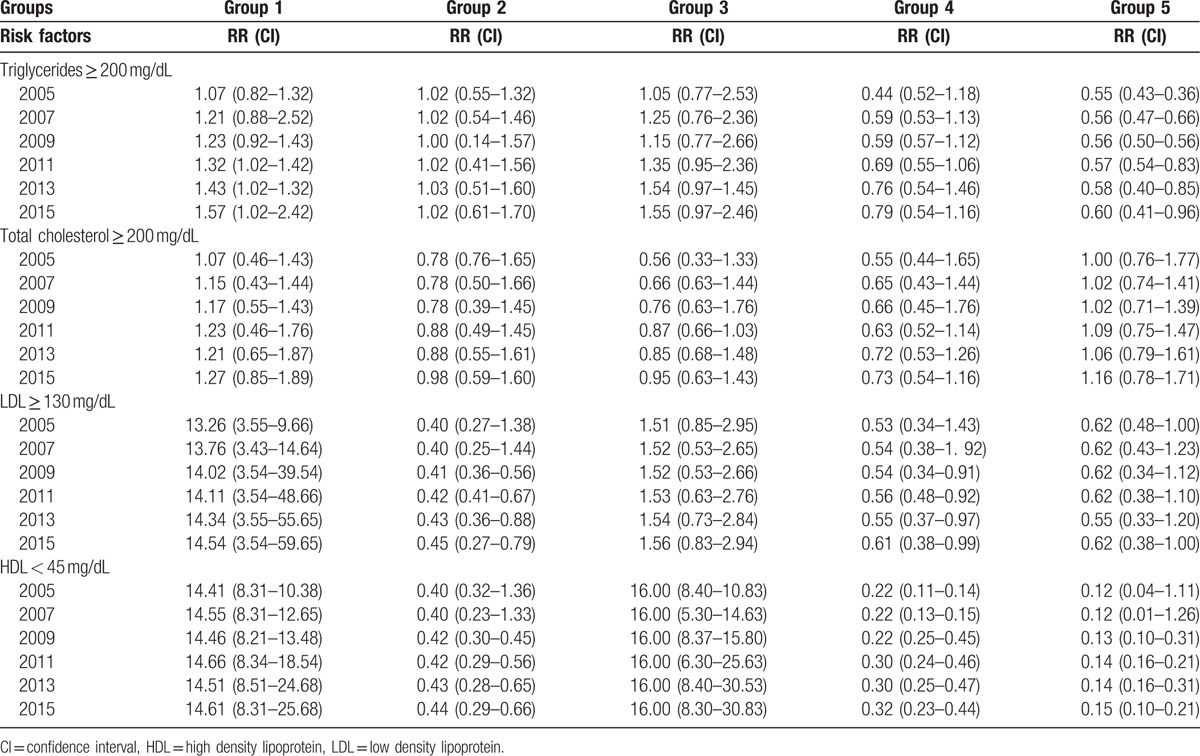
The relative risks of lipid disorders in 5 groups (*P* < .05).

## 4 Discussion

According to our findings, psychological stress was a risk factor for increasing triglycerides, and LDL and for decreasing HDL. After multinomial logistic regression they had significant differences.

Stressful situations are hazards for lipid profiles. These hazards include physical and psychological stress such as night shift work.^[18,19]^ Psychological stress had effects on different part of human body especially some organs and physiological parameters, lipid profiles are one of these parameters. Physical stresses induced by heavy physical works could affects lipid profiles too.

It seems that psychological stresses that were mentioned in many studies were more prominent in relation to dyslipidemias. In this study researcher showed that at the beginning of the study mean of triglycerides in group 5, and total cholesterol and LDL in group 3 were more than other groups. The least HDL was found in group 5. The means of lipid profiles were in the normal ranges. The highest mean related to age and BMI were observed in group 4 and 5. Other studies had demonstrated the effectiveness of wellbeing and preventive methods on lipid profiles.^[15,16]^

The older workers had dyslipidemias, they were in group 4 and 5 more than other groups. Lipid disorders were more prevalent by aging. The highest numbers of people with BMI equal and more than 30 kg/m^2^ were in group 4 and 5 too. Obesity was a risk factor for lipid disorders.^[2,3,11]^ The number of people with triglycerides more 200 mg/dL was more in group 5. With regard to cholesterol concentration, the number of people with total cholesterol more 200 was highest in group 4, the highest amount of LDL were observed in group 4 and 5, and the least amount of HDL was found in group 5.

The effects of lifestyle on blood lipid profiles had been demonstrated in other studies.^[11]^

After deleting the effects of BMI and age, the risk of increased triglycerides, and LDL were observed in group 1 that had mild psychological stress. The risk of decrease in HDL was also discovered in group 1 and 3. The group 3 had mild psychological stress with mild physical work or mild physical stress. It seems that psychological stress had more prominent effects on the lipid profiles. With moderate to heavy physical work the risk of lipid disorders were reduced. The risk of dyslipidemias could be reduced with proper nutrition and wellbeing.

Psychological stress must be assessed in all the situations especially in work environment. There were some studies that evaluated psychological stresses.^[26]^

According to the results of this study, researcher believes that job analysis and determining the risk factors for different jobs specially in works with psychological stress are necessary. Researcher demonstrated the effects of Job stress on cardiovascular risk factors in male workers.^[29]^ In other studies were worked on some specific jobs with physical and psychological risks for example shift workers and their effects on risk factors of cardiovascular disorders.^[30–32]^

In this study after deleting BMI effect or obesity and age with regression, the risks of dyslipidemias were observed in group 1 and 3; the participants who had mild psychological stress and those with mild psychological stress with mild physical activity. Another scientist studied about the burnout syndrome that could be a predictor of hyperlipidemia among employees.^[33]^ Burnout syndrome was an occupational psychological stress. Job stress could be seen in various forms which varied in different occupations.^[34]^ Working in the environment with psychological stress without a proper physical health and normal activity could be caused some disorders specially cardiovascular disorders.^[35]^

Author found that the psychological stress was an important risk factor for dyslipidemia especially in people who have worked. The modification of psychological stresses are not always possible but person's nutrition and physical activity could be modified to prevent dyslipidemias and cardiovascular disorders. Other studies had also showed the risk factors for dyslipidemias such as obesity.^[36]^

Suitable physical activity help to reduce weight and BMI resulted to improving dyslipidemias. Psychological stress is a strong risk factor for dyslipidemias. Changing this situation in daily environment and work place is necessary. One study demonstrated the effect of prevention on improving dyslipidemias.^[37]^

In other study was demonstrated the emotional effects on wellbeing of office workers.^[38]^

In this research there were not have exact job analysis for other occupational hazards and it was a limitation for this study. The author of this article recommended to the people with psychological stress to have a regular physical activity in the daily program and modifying the psychological stress by consultation with a psychologist. Job stress or chronic stress had unsuitable effects on workers’ health and occupational medicine specialist must be had attention to this.^[39,40]^

Psychological stress could be resulted from personal conflict, social and family problems, and working. Considering the importance of mental health on wellbeing, the author recommends the job modification in working situations.

## 5 Conclusions

Psychological stress was a risk factor for lipid disorders, and proper physical activity was protective in this situation. One of the physical activities is work activity; work activity without stress could be harmless and useful. However, psychological stress could be eliminated in the workplace.

## Acknowledgments

The author appreciated the supports of Mashhad University of Medical Sciences. Author thanks a lot the honorable journal and the publisher.
